# Package of NDV-Pseudotyped HIV-Luc Virus and Its Application in the Neutralization Assay for NDV Infection

**DOI:** 10.1371/journal.pone.0099905

**Published:** 2014-06-17

**Authors:** Bin Wang, Bin Wang, Peixin Liu, Tao Li, Wei Si, Jinsheng Xiu, Henggui Liu

**Affiliations:** 1 State Key Laboratory of Veterinary Biotechnology, Harbin Veterinary Research Institute, the Chinese Academy of Agricultural Sciences, Harbin, China; 2 College of Animal Sciences, Fujian Agriculture and Forestry University, Fuzhou, China; The University of Hong Kong, Hong Kong

## Abstract

Newcastle disease virus (NDV) is a member of the Paramyxovirinae subfamily and can infect most species of birds. It has been a great threat for the poultry industry all around the world. In this report, we successfully produced infectious pseudotyped pNL4-3-Luc-R^−^E^−^ (HIV-Luc) viruses with the HN and F envelope proteins of NDV. Further investigation revealed the cytoplasmic domains of HN and F, especially HN, plays a significant role in the infection efficiency of these pseudotyped HIV-Luc viruses. Replacement of, or direct fusion to the cytoplasmic domain of the HN protein by that of vesicular stomatitis virus G (VSV-G) could greatly enhance or destroy the infective potential of HN and F-pseudotyped (NDV-pseudotyped) HIV-Luc virus. We further established a novel neutralization assay to evaluate neutralizing antibodies against NDV with the NDV-pseudotyped HIV-Luc viruses. Comparative neutralization data indicate that the results determined by using the NDV-pseudotyped HIV-Luc viruses are as reliable as those by the conventional virus-neutralization assay (VN test) with native NDV. Moreover, the results show that the novel neutralization assay is more sensitive than the VN test.

## Introduction

Newcastle disease virus (NDV) belongs to the Paramyxoviridae family. It has been found worldwide since its first report among poultry in the areas of Java, Indonesia, and England in 1926. This virus has an extensive range of susceptible hosts, with 27 of the 50 orders of birds reported to be capable of infection by NDV [1], making Newcastle disease (ND) a global animal health concern. Although the vaccination of chickens is performed throughout the world, this disease still remains endemic in poultry in many regions. According to the severity of disease in chickens, isolates of NDV can be categorized into three pathotypes: the lentogenic (avirulent), mesogenic (intermediate virulence), and velogenic (highly virulent) strains. Infections with velogenic strains have had devastating effects on the poultry industry due to the high rates of morbidity and mortality. Thus, virulent NDV isolates are notifiable to the Office of International Epizootes (OIE) with obligatory control measures upon the occurrence of outbreaks of the disease.

The genome of NDV is a nonsegmented, single-stranded, negative-sense RNA and encodes six viral proteins from six genes: nucleocapsid (NP), phosphoprotein (P), matrix (M), fusion (F), hemagglutinin-neuraminidase (HN) and large RNA-dependent RNA polymerase (L) proteins [2]. Two additional proteins, V and W, are produced by RNA editing during P gene transcription [3]. HN and F are membrane proteins of NDV that are responsible for mediating NDV infection. While HN mediates viral attachment to the sialic acid receptors on the cells, F protein directs membrane fusion between the virus and cells [4], [5]. Antibodies against HN and F have shown the ability to block the receptor binding and virus-cell fusion, respectively, thereby protecting birds from virulent NDV infection [6]–[11]. The elicitation of neutralizing antibodies is therefore considered a high priority of vaccine design. Moreover, having effective assays that evaluate these neutralizing antibodies are necessary for assessing viral infection and vaccine efficacy. At present, the haemagglutination inhibition (HI) and the virus-neutralization (VN) assays are available for the detection of neutralizing antibodies against NDV [12]–[16]. While the HI assay is able to measure only the neutralizing antibodies against the receptor-binding site and has limitations in terms of its low sensitivity and high incidence of false-positives [17]–[19], the conventionally used VN assay is labor-intensive, time-consuming (requiring a minimum of 4 days), and less objective [15], making it unsuitable for large scale evaluation of neutralizing antibodies. Pseudovirus-based neutralization assays, however, have been proven to be a rapid, sensitive, and specific high-throughput system for the evaluation of neutralizing antibodies and antiviral drug discovery. The pseudovirus backbone (commonly a lentiviral or retroviral vector) generally carries a reporter gene, such as luciferase, in which the neutralizing ability of antibodies can be easily quantified. Pseudotyped viral particles by heterologous viral glycoproteins have been described for several viruses, including influenza virus [20], [21], SARS coronavirus [22], Sendai virus [23] and hepatitis C virus [24]. However, pseudotyped viruses with the NDV envelope glycoproteins HN and F have not yet been reported. Here, we report on the successful production of HN and F-pseudotyped (NDV-pseudotyped) HIV-Luc viral particles and the factors impacting the infection efficiency of NDV-pseudoviruses. We further established a novel neutralization assay with the NDV-pseudotyped HIV-Luc viruses to evaluate neutralizing antibodies against NDV.

## Materials and Methods

### Cell Line, Virus and Monoclonal Antibodies

293 T cells were used for infection and cultured in Dulbecco’s Modified Eagle Medium (DMEM) supplemented with 10% heat-inactivated fetal bovine serum (FBS, Hyclone) in a humidified incubator at 37°C under an atmosphere of 5% CO_2_. Genotype VII NDV strain (isolated in our lab) was propagated in the allantoic cavities of 9- to 10-day old specific pathogen free (SPF) embryonated chicken eggs and kept at −20°C for RNA extraction and neutralization assay. Monoclonal antibodies (mAbs) against HN and F were a kind gift from Dr. Shunlin Hu (the University of Yang Zhou).

### Plasmid Construction

Total RNA was extracted from NDV using a commercial RNA extraction kit (Qiagen), and used for cDNA production with random primers. Wild-type HN and F were amplified from cDNA templates with the primer pairs: HN-U (ATCAGAATTCATGGACCGCGCGGTTAACAG)/HN-L (CGGCTCGAGTTAAACTCTATCATCCTTG) and F-U (GATGTAAGCTTGTAATGGGCTCCAAACCTTCTACC)/F-L (GATGTTCTAGATCATGCTCTTGCAGTGGCTCTCAT), respectively. The PCR products were inserted into the pCAGGS vector between the *EcoR* Ι-*Xho* I and *Hind* III -*Xba* I sites to generate pCAGGS-HN and pCAGGS-F, respectively. The mutant HN and F were constructed as the following strategy: VCT/HN and F/VCT were generated by replacement of the cytoplasmic domains of HN and F with the cytoplasmic domain of VSV-G (VCT); VCT-HN was constructed by directly fusing VCT with the start codon ATG at the N-terminus to the N-terminus of HN; FΔCT mutant was constructed by deleting the cytoplasmic domain of F. All wild-type and mutant HN and F were inserted into pCAGGS to facilitate their expression in 293 T cells.

### Flow Cytometry Analysis

293 T cells expressing the wild-type or mutant HN and F proteins of NDV were resuspended in phosphate buffered saline (PBS). 1×10^6^ cells in 100 µl PBS were stained with monoclonal antibodies against HN or F for 30 min at room temperature. Unbound antibodies were removed by washing three times. The cells were further labelled with FITC-conjugated rabbit anti-mouse IgG second antibodies (Invitrogen) for 30 min and washed as previously described and finally fixed with 2% polyformaldehyde. The data were collected from 1×10^4^ cells in FACS and analyzed using FlowJo software.

### Production of Pseudotyped Virus Particles

Production of HIV-Luc viral particles pseudotyped with NDV envelope proteins or VSV-G were performed as previously described [25]. Briefly, 293 T cells in 10 cm diameter dishes were transfected with various combinations of wild-type or mutant HN and F plus the lentiviral vector HIV-Luc (8 µg each) using HiTrans transfection reagent (Jpson Biosciences). The supernatant of cultured cells was replaced with fresh medium 4 h post transfection to rule out the influence of existing complexes of plasmid-transfection reagent on the following pseudovirus infection. After incubation for 48–72 h, the supernatants of transfected cells containing pseudotyped viruses were harvested, filtered through a 0.45 µm syringe filter, and the pseudotyped viruses were used to infect 293 T cells immediately or frozen at −80°C. If required, pseudotyped viruses were purified by ultracentrifugation at 28000 rpm in a SW32Ti rotor (Beckman) with a 20% sucrose cushion for 3 h at 4°C. The pellets were resuspended in PBS and aliquoted for storage at −80°C. All pseudotyped viruses produced as described above were titrated using a p24 antigen capture assay (Key-Bio).

### ELISA Assay

ELISA assay was performed as previously described [26]. Briefly, purified pseudotyped viruses were immobilized on ELISA plate (Thermo fisher Scientific) in 100 µl 0.05 M carbonate-bicarbonate (pH 9.6) by overnight incubation at 4°C. The plate was washed four times with PBS containing 0.05% Tween 20 (PBST) and blocked with 5% skim milk in PBST at 37°C for 1 h. The plate was washed again as above and the first detecting monoclonal antibody was added to each well. After incubation at 37°C for 1 h, the plate was washed four times with PBST before the addition of horseradish peroxidase (HRP)-conjugated goat anti-mouse IgG (Invitrogen) to each well and incubation at 37°C for 1 h. The plate was washed again as above, 100 µl TMB substrate was added to each well. The reaction was stopped using 0.5 N H_2_SO_4_ after incubation at room temperature for 30 min. The OD_450_ value of each well was immediately read with a microplate photometer (ELx800, BioTek).

### Pseudotyped Virus Infection and Normalization

293 T cells (1×10^4^ cells/well) were seeded to 96-well plates and infected with 100 µl of the pseudotyped viruses the next day. After incubation for 1 h, pseudotyped virus containing supernatant was removed and replaced with fresh DMEM supplemented with 10% FBS. 48 h post-infection, 293 T cells were lysed with 50 ul lysis buffer (Promega). Relative luminescence Units (RLU) of luciferase activity in 293 T cells was detected using the Bright-Glo Luciferase Assay System (Promega) with PB12 Luminometer (Berthold) and normalized by the following formula: (luciferase activity of cells/p24 titer of the pseudotyped virus) ×100.

### Titration of NDV and NDV-Pseudotyped HIV-Luc Virus with Tissue Culture Infectious dose 50 (TCID_50_) Assay

NDV was titrated as previously described with minimal modifications [27]. In brief, DF-1 cells were seeded in 96-well plates with 1×10^5^ cells per well. After 24 h, virus samples were ten-fold serially diluted with DMEM containing 2% FBS. The DF-1 cells were infected with 100 µl of the diluted virus samples and incubated at 37°C for 4 days. The cytopathogenic effect (CPE) on cells in each well was observed using light microscopy. The TCID_50_ values were calculated by the Reed-Müench method [28]. Titration of the NDV-pseudotyped HIV-Luc viruses was performed similarly. Briefly, 293 T cells were seeded in 96-well plates with 2×10^4^ cells per well and infected with ten-fold serially diluted NDV-pseudotyped HIV-Luc viruses. The pseudotyped virus infection was detected by measuring the luciferase activity in the cells 48 h post infection. The TCID_50_ values were calculated by the Reed-Müench method.

### Neutralization Assay with NDV-Pseudotyped HIV-Luc Virus

Immune sera against NDV or field sera were heat-inactivated at 56°C for 30 min, two-fold serially diluted and mixed with 100 TCID_50_ of NDV-pseudotyped HIV-Luc viruses. After incubation for 1 h at room temperature, the pseudotyped-virus-serum mixtures were transferred to 293 T cells pre-plated in 96-well plates (10^4^ cells/well) with 2 to 4 wells per dilution. After incubation at 37°C under an atmosphere of 5% CO_2_ for 48 h, luciferase activity in the cells was detected as described above. Background luminescence produced by cell-only controls (no pseudotyped virus) and positive luminescence produced by negative serum control (negative serum plus pseudotyped viruses) were included. The neutralization efficiency was calculated with the following formula: (1- (luciferase activity in experimental well - luciferase activity in cell-only control)/(luciferase activity in negative serum control - luciferase activity in cell-only control)) ×100%. The serum dilution at 50% inhibition was calculated using GraphPad Prism version 5 software and set as neutralization titer (NT titer) as previously described [26].

### Conventional Neutralization Assay

The conventional neutralization assay was performed with DF-1 cells as previously described [16]. Briefly, the heat-inactivated sera were two-fold serially diluted and incubated with an equal volume of NDV (100 µl containing 100 TCID_50_) at 37°C for 1 h. The serum-NDV mixtures were then added to DF-1 cell monolayers on a 96-well plate with 4 wells per dilution. After 4 days of incubation, the neutralization was determined by CPE observation. The highest dilution of serum at which infectivity was inhibited in 50% of the wells was set as NT titer as previously described [26].

### Statistical Analysis

All statistical analyses were performed using the paired one-tailed Student’s *t* test as previously described [29]. *P* values less than 0.05 were considered statistically significant. Results were presented as mean values ± the standard deviation (SD) of at least three independent experiments.

## Results

### Construction and Expression of Wild-type and Mutant HN and F of NDV

**Figure 1:**
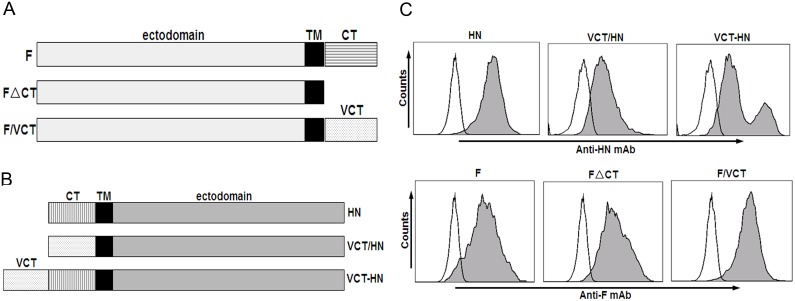
Schematic organization and protein expression of wild-type and mutant HN and F proteins. (A) and (B) Schematic representation of wild-type and mutant F and HN proteins. CT: cytoplasmic domain; TM: transmembrane domain; VCT: cytoplasmic domain of VSV-G protein ranging from 491–511 aa; (C) Flow cytometric analysis of cell surface expression of wild-type and mutant HN and F proteins. Individual plasmid of wild-type and mutant HN or F was transfected into 293 T cells. 24 h post transfection, protein expression was evaluated with FACS after staining with anti-HN mAb (upper panels of histograms) or anti-F mAb (lower panels of histograms).

In this research, we constructed six recombinant plasmids carrying wild-type F (F); FΔCT, in which the cytoplasmic domain of the F protein (amino residue 526–553) was deleted; F/VCT, in which the cytoplasmic domain of the F protein was replaced by VCT (amino residue 491–511 of VSV-G); wild-type HN (HN); VCT/HN, in which the cytoplasmic domain of HN (amino residue 1–23) was replaced by VCT; and VCT-HN, in which VCT was directly fused to the HN at the N-terminus, respectively (Figure 1A and B). The expression of the wild-type and mutant HN and F proteins was confirmed by transfection of the individual recombinant plasmids into 293 T cells followed by FACS analysis 24 h later. As shown in figure 1C, HN and F proteins from each of the recombinant plasmids were highly expressed on 293 T cells.

### Production of NDV-Pseudotyped HIV-Luc Viral Particles

**Figure 2:**
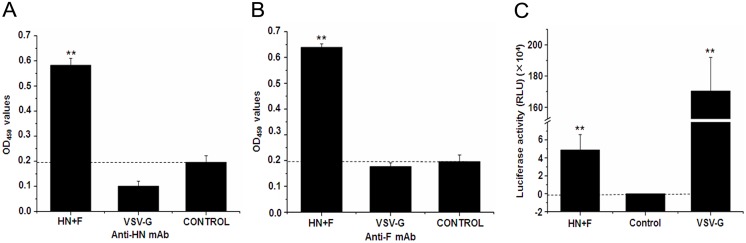
Analysis of NDV-pseudotyped HIV-Luc viruses. HIV-Luc vector was co-transfected into 293 T cells with HN and F or VSV-G. Pseudoviruses were harvested 48 h post transfection and used for the analysis of their incorporation and infection. (A) and (B) The pseudotyped HIV-Luc viruses by HN and F or VSV-G envelope proteins were purified with ultracentrifugation and coated to the ELISA plate. F and HN proteins incorporated into pseudotyped HIV-Luc virus were probed by anti-HN mAb (A) or anti-F mAb (B); (C) The pseudotyped HIV-Luc viruses by HN and F or VSV-G envelope proteins were used to infect 293 T cells in 96-well plates. 48 h later, luciferase activity of infected cells was detected and normalized with titer of p24 protein in pseudoviruses. HN + F: NDV-pseudotyped HIV-Luc virus; VSV-G: VSV-G-pseudotyped HIV-Luc virus; control: uninfected cells. All results are shown as means ± SD from three independent experiments. “**” indicated *P*<0.01.

HIV-Luc is an HIV-1 based lentiviral vector bearing the luciferase reporter gene and has been used extensively in the production of pseudotyped viruses [30]–[32]. To determine whether the HN and F proteins of NDV could be incorporated into pseudotyped HIV-Luc viral particles, they were co-transfected into 293 T cells with the HIV-Luc vector. Pseudotyped viruses in the supernatant were purified using ultracentrifugation and the incorporation of HN and F was determined by ELISA assay. As shown in figure 2A and B, probing with anti-HN or -F mAbs showed significantly higher OD_450_ values in the wells coated with NDV-pseudotyped HIV-Luc viruses than in either the control or VSV-G-pseudotyped viral particles (*P*<0.01). These results indicated the successful incorporation of HN and F into pseudotyped viral particles. We next looked to address whether the NDV-pseudotyped viruses was infectious in 293 T cells. As shown in figure 2C, luciferase activity in the cells infected with NDV-pseudotyped HIV-Luc viruses reached more than 40000 RLU, much higher than in the control (*P*<0.01), indicating the successful production of an infectious NDV-pseudotyped HIV-Luc virus.

### Factors Affecting the Infection Efficiency of NDV-Pseudotyped HIV-Luc Virus

**Figure 3:**
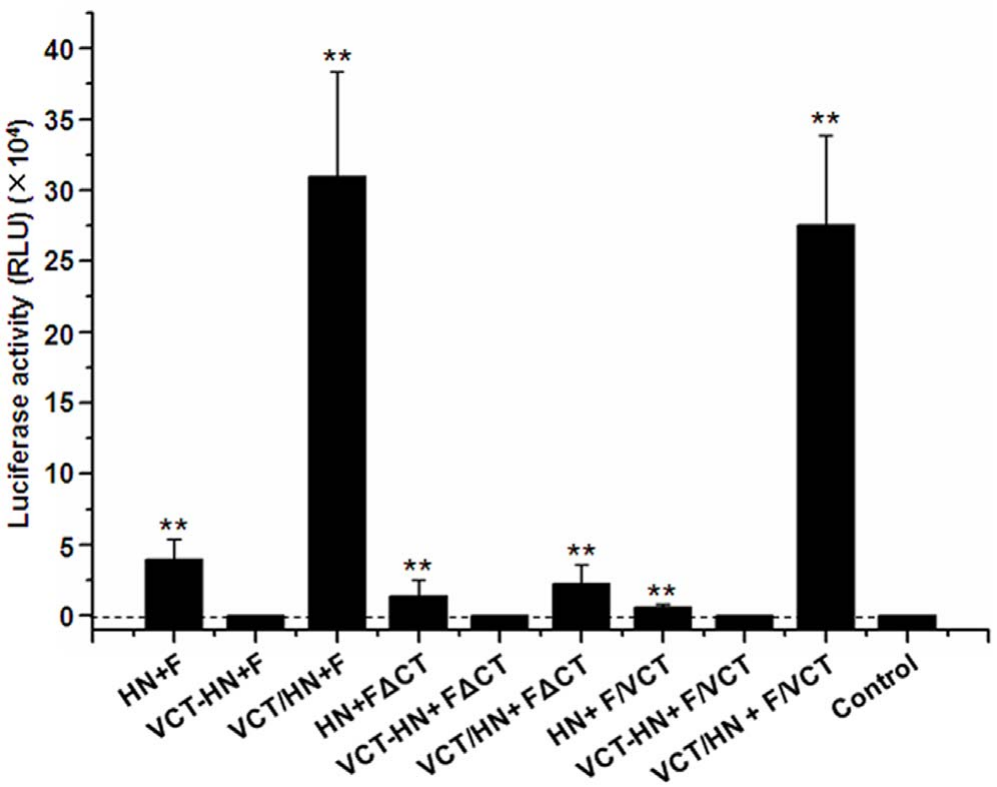
Analysis of factors impacting the infection efficiency of NDV-pseudotyped virus. A panel of mutant and wild-type HN and F combinations were co-transfected into 293 T cells with the HIV-Luc vector. Pseudoviruses in the culture medium were harvested 48 h later and used to infect 293 T cells in 96-well plates. The luciferase activity of the infected cells was detected 48 h post infection and normalized with the titer of p24 in the pseudoviruses. All results are shown as means ± SD from four independent experiments. “**” indicated *P*<0.01.

Although the NDV-pseudotyped HIV-Luc viral particle was successfully produced, the infection efficiency was found to be over 34-fold lower than with the VSV-G-pseudotyped HIV-Luc virus (*P*<0.01) (Figure 2C). We hypothesized that this may be due to the impact of the cytoplasmic domain of the envelope proteins [33], [34]. To address this, four plasmids containing mutant HN or F were constructed (Figure 1A and B). After confirming their expression on 293 T cells (Figure 1C), nine combinations of wild-type and mutant HN and F were co-transfected into 293 T cells with HIV-Luc vector to produce corresponding pseudotyped viruses. As shown in figure 3, of the nine combinations, six produce infectious pseudotyped viruses including HN plus F, HN plus FΔCT, HN plus F/VCT, VCT/HN plus F, VCT/HN plus FΔCT and VCT/HN plus F/VCT. Among the infectious pseudotyped viruses, the infection efficiencies of VCT/HN plus F and VCT/HN plus F/VCT were the highest, reaching from 8 to 50 folds higher than all other groups (*P*<0.01). The infection efficiency between the two combinations was not significantly different (*P*>0.05). This indicated that replacement of the cytoplasmic domain of HN by VCT greatly enhanced the infection efficiency of NDV-pseudotyped HIV-Luc viruses. However, fusion of VCT to the N-terminus of the cytoplasmic domain of HN resulted in the complete loss of infectivity as shown in the combinations of VCT-HN plus F, VCT-HN plus FΔCT and VCT-HN plus F/VCT (Figure 3). These results revealed the important role of the cytoplasmic domain of HN on the infection efficiency of NDV-pseudotyped HIV-Luc viruses. Moreover, when we compared VCT/HN plus FΔCT with the combinations of VCT/HN plus F and VCT/HN plus F/VCT, we saw a significant reduction in the infection efficiency with the pseudotyped virus containing FΔCT (*P*<0.01). This result revealed that the cytoplasmic domain of F also plays an important role in the infection efficiency of NDV-pseudotyped HIV-Luc viruses. This was further confirmed by comparison among the combinations of HN plus F, HN plus FΔCT and HN plus F/VCT, where both the deletion and VCT replacement of the cytoplasmic domain of F significantly reduced infectivity (*P*<0.01) (Figure 3). Altogether, these results indicated that the cytoplasmic domain of HN was the dominant factor behind the infection efficiency of the NDV-pseudotyped HIV-Luc virus, and VCT/HN plus F or F/VCT is the optimal recombination for the production of highly infectious pseudoviruses.

### Development of a Neutralization Assay with NDV-Pseudotyped HIV-Luc Virus

**Figure 4:**
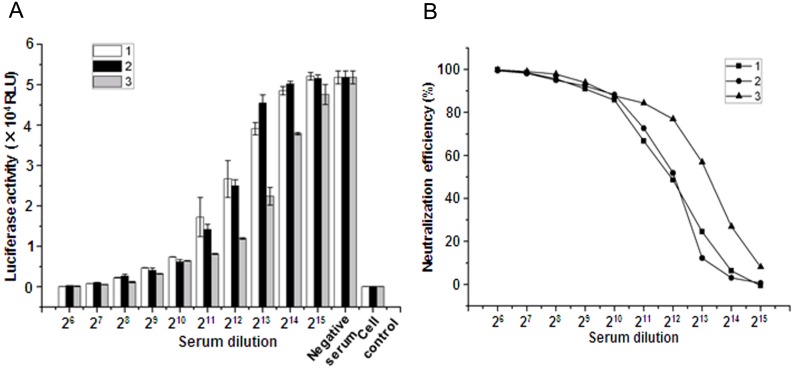
Neutralization assay of NDV-pseudotyped HIV-Luc virus by the anti-NDV sera. Sera against NDV were two-fold serially diluted with DMEM. 100 µl titrated NDV-pseudotyped HIV-Luc viruses were mixed with an equal volume of diluted anti-NDV sera. The mixtures were added to 293 T cells in 96-well plates after incubating at room temperature for 1 h. (A) Neutralization of NDV-pseudotyped virus by the immune sera against NDV. Negative serum and cell control refer to the cells infected with a mixture of negative serum from chickens with NDV-pseudotyped virus and cells without pseudovirus infection, respectively; (B) Neutralization efficiency of NDV-pseudotyped HIV-Luc virus by the three anti-NDV sera at an indicated dilution. Results are shown as means ± SD from four independent infections.

To determine the feasibility of measuring neutralizing antibodies against NDV using the NDV-pseudotyped HIV-Luc virus, three immune sera against NDV were two-fold serially diluted and incubated with an equal volume of VCT/HN and F/VCT-pseudotyped HIV-Luc viruses containing 100 TCID_50_ for 1 h at room temperature. The serum and pseudovirus mixtures were then used to infect 293 T cells in 96-well plates for 48 h. As shown in figure 4, luciferase activity increased along with serum dilutions (Figure 4A). Accordingly, the neutralization efficiency calculated based on the RLU of luciferase activity, corresponded negatively with serum dilutions (Figure 4B). There was a dose-dependent correlation between the neutralization efficiency and serum dilutions. These results confirmed the successful use of the NDV-pseudotyped HIV-Luc virus-based system for measuring neutralizing antibodies against NDV.

### Validation of the Neutralization Assay with NDV-Pseudotyped HIV-Luc Virus

**Figure 5:**
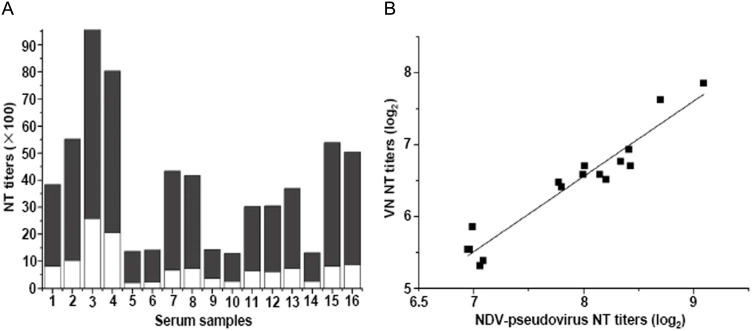
Comparison of NT titers determined by NDV-pseudotyped HIV-Luc virus with those by VN test. (A) NT titers of sixteen immune sera against NDV were determined by NDV-pseudotyped HIV-Luc viruses and VN test, respectively. The obtained data were stacked on the column. White and black column were NT titers determined by VN test and NDV-pseudotyped HIV-Luc viruses, respectively. (B) Correlation between NT titers determined by NDV-pseudotyped HIV-Luc virus and those obtained with VN test. The correlation coefficient was 0.92. NT titer (log_2_) values determined by NDV-pseudotyped HIV-Luc virus plotted between 6.5 and 9 on the x-axis scale, whereas, those by VN test plotted between 5 and 8 on the y-axis scale.

To validate the neutralization assay with NDV-pseudotyped HIV-Luc viruses, we compared the NT titers determined by the NDV-pseudotyped HIV-Luc virus-based neutralization assay with those measured by a reference neutralization assay (VN test). As shown in figure 5, NT titers from each of the sixteen immune sera against NDV were successfully detected by both neutralization assays. Moreover, the NT titers determined by the NDV-pseudotyped HIV-Luc virus-based neutralization assay were two to five folds higher than those obtained with the conventional VN test, ranging from 1000 to 9000 and 200 to 3000, respectively (Figure 5A). There was a strong correlation between the two neutralization assays (y = 1.05x−1.81, R^2^ = 0.92), showing that the NDV-pseudotyped HIV-Luc virus-based neutralization assay was as reliable as the conventional VN test (Figure 5B). We further performed the neutralization assay with NDV-pseudotyped HIV-Luc virus and conventional VN test on sixteen field sera which were all positive for NDV antigens with ELISA assay (data not shown). As shown at table 1, neutralizing antibodies were successfully detected in all sixteen sera using the NDV-pseudotyped HIV-Luc virus versus twelve with the VN test. Our results indicate that the NDV-pseudotyped HIV-Luc virus-based neutralization assay was more sensitive than the conventional VN test.

**Table 1:** Comparison of the results of the neutralization assays with NDV-pseudotyped HIV-Luc viruses and VN test.

Neutralization assays	No. of samples with neutralizingantibodies (positive/total)	No. of samples without neutralizingantibodies (negative/total)	NT positiverate (%)
VN test	12/16*	4/16*	75
NDV-pseudovirus	16/16*	0/16*	100

Note: *indicates that the samples were positive for NDV antigens with ELISA assay.

## Discussion

Pseudotyping provides the ability to introduce proteins from different sources into a viral vector shell [35]. Many pseudotyped vectors have been developed using retroviruses or lentiviruses, such as the Mo-MuLV vector from Moloney murine leukemia virus and HIV-1 vector from the HIV-1 virus [36], [37]. Given that some components essential for viral replication or persistent infection have been deleted from the genome, pseudotyped viruses can only take on a single-cycle of infection. Thus, they are a safe alternative for use in the research lab with extremely virulent viruses. So far, pseudoviruses have been used for the identification of virus receptors, screening and evaluation of neutralizing antibodies, transduction of genes to target cells and exploration of the mechanisms of virus infection [38]–[44]. Previously, envelope proteins of Sendai virus, a member of paramyxovirus, have been reported to successfully pseudotype the lentivirus vector derived from simian immunodeficiency virus [23]. Recently, two other paramyxovirus pseudotyped lentiviral vectors have been successfully reported, establishing the ability of paramyxoviruses to form successful pseudoviruses [45], [46]. Here, we successfully produced pseudotyped HIV-Luc virus particles with the HN and F proteins of NDV (Figure 2C). This is the first report of a NDV-pseudotyped retroviral vector and further adds to the list of paramyxovirus pseudotyped lentivrial vectors.

Previous reports have shown that the interaction between the matrix protein and the cytoplasmic domain of envelope proteins was required for the incorporation of the envelope proteins into mature viral particles [33], [34], [47]. Indeed, we saw a lower infection efficiency with the NDV-pseudotyped HIV-Luc virus compared to the VSV-G pseudotyped HIV-Luc virus (Figure 2C), which we associated with a less-optimal interaction between the HIV-1 matrix and the cytoplasmic domain of NDV envelope proteins. For paramyxoviruses, there are two envelope proteins, HN and F. Given that deletion of the cytoplasmic domain of F did not obstruct pseudoviral packaging (Figure 3), we speculated that the interaction between F and matrix protein plays a non-essential role for the packaging of the NDV-pseudotyped HIV-Luc virus. We suggest that the interaction between F and HN is sufficient for F incorporation into virons [48], [49]. However, although we were able to successfully produce a panel of functional NDV-pseudotyped HIV-Luc viruses (Figure 3), we found that some would lose their infectivity if not used immediately, such as for the pseudotyped viruses with HN plus FΔCT and HN plus F/VCT (data not shown). When the cytoplasmic domain of HN was replaced with a more optimal one (VCT), the stability of the NDV-pseudovirus substantially improved, remaining stable even after repeat thawing. These phenomena perhaps indicate that the interaction between the HIV-1 matrix protein and the cytoplasmic domains of HN and F, especially HN protein, correlates with the stabilization of the NDV-pseudotyped HIV-Luc virus. Here, we also found that replacement of the cytoplasmic domain of HN with VCT enhanced the infection efficiency and stabilization of the NDV-pseudotyped HIV-Luc virus, while the infectivity was lost when VCT was added directly to the N-terminus of the HN protein, regardless of which combination of F protein was used (Figure 3). These results differed from those seen in previous research with the Sendai virus where similar replacement and fusion were carried out [23]. These results further suggest that among the paramyxoviruses, their characterizations of packaging lentiviral vectors may differ from each other.

For paramyxoviruses, receptor binding and hemagglutinin-neurominidase active (HA) sites reside in the same region of HN [50]. Therefore, the HI assay is sometimes used for evaluating the neutralizing antibodies that recognize receptor-binging site I [51]. After NDV binding to the receptor on the cells, conformational changes in the envelope proteins will take place [52], [53]. The researches on neutralizing antibodies against HIV-1 have shown that numerous antibodies that recognize regions outside of the receptor-binding site are able to neutralize virus infection [54], [55]. For these neutralizing antibodies, the HI assay would be inadequate. More commonly, neutralizing antibodies against NDV are evaluated by VN tests with native NDV [56], [57]. However, the VN test with native NDV is time-consuming, requiring at least 4 days, and less-objective. In this study, we developed a novel assay to evaluate the neutralizing antibodies using the NDV-pseudotyped HIV-Luc virus (Figure 4 and 5). This assay is capable of evaluating all neutralization activities of antibodies through detection of viral entrance into host cells. Compared to currently available VN tests [58], the NDV-pseudotyped HIV-Luc virus-based assay only require two days, and is easily performed in large scale with a luminometer. Our results showed that the NT titers determined with the NDV-pseudotyped HIV-Luc virus-based assays were around 4 times higher than those with conventional VN tests (Figure 5A). This reflects on the greater sensitivity for detecting NT titers using our established luciferase-based assay compared with performing microscopic observations of CPE using the VN assay. The sensitivity of the pseudovirus-based assay was further confirmed in the detection of neutralizing antibodies (table 1) in the two negative sera as determined with the conventional VN test. These obvious advantages of being a high-throughput, rapid, sensitive, reproducible and less subjective system have encouraged the development of many other pseudotyped lentivirus for the establishment of neutralization assays, such as equine influenza, Nipah virus, Chikungunya Virus, Coxsackievirus A16 and HIV-1 [25], [26], [45], [59]–[61]. In this novel neutralization assay, there are two methods to titrate NDV-pseudotyped HIV-Luc viruses, one is by measuring the luciferase activity after pseudovirus infection [60], and the other is with the TCID_50_ assay. Our results showed that titration by measurement of the luciferase activity is easily performed, however, the value titrated by this method is more variable and less objective compared to that with the TCID_50_ assay. We prefer the latter method to titrate NDV-pseudotyped HIV-Luc viruses.

In summary, this study reported the successful production of HIV-Luc viruses pseudotyped with the envelope proteins of NDV. We further analyzed the factors affecting the infection efficiency of NDV-pseudotyped HIV-Luc viruses and optimized the plasmid combination to produce highly infectious pseudotyped viruses. Moreover, a neutralization assay using the NDV-pseudotyped HIV-Luc viruses was successfully developed for evaluating the neutralizing antibodies against NDV.
